# A modified version of diagonal systematic sampling in the presence of linear trend

**DOI:** 10.1371/journal.pone.0265179

**Published:** 2022-03-31

**Authors:** Muhammad Azeem

**Affiliations:** Department of Statistics, University of Malakand, Lower Dir, Khyber Pakhtunkhwa, Pakistan; IIT Madras, INDIA

## Abstract

Systematic sampling is one of the simplest and popular methods for selecting a random sample from a finite population. The diagonal systematic sampling scheme is a type of systematic sampling design which has gained the attention of researchers during the last two decades. In this paper, a modification to the conventional diagonal systematic sampling design is proposed for use in situations where population units follow a linear trend. It is found that the proposed strategy reduces the variance of the diagonal systematic sampling thus resulting in an efficient sampling design. The mathematical conditions under which the suggested modified diagonal systematic sampling design is more precise than some of the available sampling designs are derived. With the help of a numerical illustration using milk yield data, it is shown that the proposed sampling scheme is more efficient than some of the available sampling schemes.

## 1. Introduction

In survey sampling, the linear systematic sampling scheme originally developed by Madow and Madow [1] is used to obtain a sample of size *n* units from a finite population of size *N* units in such a way that the first unit is obtained from the first *k* (= *N*/*n*) units and then every *k*th unit is systematically selected in the sample. A limitation of the usual linear systematic sampling procedure is that it requires the population size to be a constant multiple of the required sample size. To cope with this issue, Lahiri [2] introduced circular systematic sampling scheme. Mukerjee and Sengupta [3] proposed some optimal sampling strategies for estimation of mean. Chang and Huang [4] developed a modified version of the systematic sampling popularly known as the remainder systematic sampling for use in situations where the population size is not a constant multiple of the required sample size. The concept of diagonal systematic sampling scheme as an alternative approach to the classical systematic sampling was introduced by Subramani [5]. Sampath and Varalakshmi [6] introduced a new modified systematic sampling method known as diagonal circular systematic sampling scheme. Subramani [7] developed a generalized form of the original diagonal systematic sampling scheme. Another modified version of the linear systematic sampling for use in situations where the sample size is odd, was suggested by Subramani [8] which was found to be more efficient than linear systematic sampling scheme. Khan et al. [9] presented a modified version of systematic sampling under equal probability sampling which was a generalization of both linear and circular systematic sampling schemes. A generalization of the usual systematic sampling method was suggested by Subramani and Gupta [10] which improved the linear systematic sampling in terms of efficiency. The method suggested by Subramani and Gupta [10] was practically more useful than linear systematic sampling as it didn’t require the population size to be a constant multiple of the required sample size. However, the limitation of the Subramani and Gupta [10] method was that the sample mean based on this sampling scheme was a biased estimator of the mean of finite population. Subramani and Singh [11] developed an optimal form of circular systematic sampling for populations following linear trend. Naidoo et al. [12] developed a new modified version of balanced systematic sampling. A comparative performance of circular systematic and simple random sampling under linear trend scenario was studied by Subramani [13]. Gupta et al. [14] introduced a new modification of systematic sampling which is based on multiple random starts. Recently, Azeem et al. [15] proposed a new systematic sampling design for estimation of population mean. Further studies related to systematic sampling can be found in Madow [16], Yates [17], Bellhouse and Rao [18], Bellhouse [19], Fountain and Pathak [20], Sampath and Uthayakumaran [21], Subramani [22], Khan et al. [23], and Naidoo et al. [24] etc.

In this paper, an efficient modified diagonal systematic sampling method is proposed for situations where the population units follow a linear trend. The mathematical conditions under which the suggested sampling design is more efficient than some of the existing sampling schemes have been derived. It has been shown that the new systematic sampling scheme is more precise than some of the existing sampling schemes.

## 2. Proposed modified diagonal systematic sampling scheme

Let the population consists of *N* units with labels 1, 2, 3, …, *N* and it is required to draw a sample of size *n* such that *N* = *nk* = (*n* − 1)*k* + *k*. Motivated by Subramani [8], a modified version of diagonal systematic sampling is proposed. While the conventional diagonal systematic sampling selects a sample of size *n* from the whole population regarded as a single group, the proposed method separates the last *k* units from the rest of the population. Thus the population is divided into two groups where the first *n*−1 units of the required sample are selected from the first group and the last unit is selected independently from the second group. Such a partition of the population into two disjoint groups results in a more efficient sampling scheme as shown in section 5 and 6. The steps involved in the proposed method are as follows:

1. Partition the population into two sets: Set-1 and Set-2, in such a manner that Set-1 receives the first (*n* − 1)*k* units and Set-2 receives the remaining *k* units.
2. In Set-1, arrange the units in a (*n* − 1) × *k* square matrix. In Set-2, arrange the *k* units in a row having units *y*_(*n*−1)*k*+1_, *y*_(*n*−1)*k*+2_, …, *y*_*nk*_ as shown in Table 1.
3. Obtain two random numbers *r*_1_ and *r*_2_ where 1 ≤ *r*_1_ ≤ *k* and 1 ≤ *r*_2_ ≤ *k*. In Set-1, the units are drawn in such a way that the selected *n* − 1 units are the entries in the diagonal or broken diagonal of the matrix. In Set-2, a unit is selected at random and is combined with the *n* − 1 units selected from Set-1 to complete the sample of size *n*.

**Table 1:** Layout of the population units.

Set-1					Set-2				
S.No.	1	2	…	*k*	S.No.	1	2	…	*k*
1	y_1_	y_2_	…	*y*_*k*_					
2	*y*_*k*+1_	*y*_*k*+2_	…	*y*_2*k*_					
3	*y*_2*k*+1_	*y*_2*k*+2_	…	*y*_3*k*_					
…	…	…	…	…					
*n* − 1	*y*_(*n*−2)*k*+1_	*y*_(*n*−2)*k*+2_	…	*y*_(*n*−1)*k*_	*n*	*y*_(*n*−1)*k*+1_	*y*_(*n*−1)*k*+2_	…	*y*_*nk*_

It is clear that the suggested modified diagonal systematic sampling design has *k* × *k* = *k*^2^ possible samples each of size *n*. For the proposed method, the first and second order probabilities of inclusion are:

$$\pi_{i}=\frac{1}{k},$$ (1)

and

$$\pi_{ij}=\{\begin{array}{ll} \frac{1}{k} & \text{if}\;i\text{th}\;\text{and}\;j\text{th}\;\text{units}\;\text{are}\;\text{from}\;\text{the}\;\text{same}\;\text{diagonal}\;\text{or}\;\text{broken}\;\text{diagonal}\;\text{of}\;\text{Set-1,}\\ \frac{1}{k^{2}} & \text{if}\;i\text{th}\;\text{and}\;j\text{th}\;\text{units}\;\text{are}\;\text{from}\;\text{Set-1}\;\text{and}\;\text{Set-2}\;\text{respectively,}\\ \text{0} & \text{otherwise}. \end{array}$$ (2)

Generally, the selected sampling units are:

$$\begin{array}{ll} S_{r_{1}r_{2}}=\left\{y_{r_{1}},y_{\left(k+1\right)+r_{1}},\ldots ,\;y_{\left(n-2\right)\left(k+1\right)+r_{1}},y_{\left(n-1\right)k+r_{2}}\right\}, & \text{if}\;r_{1}\leq k-n+2,\\ S_{r_{1}r_{2}}=\left\{y_{r_{1}},y_{\left(k+1\right)+r_{1}},\ldots ,\;y_{t\left(k+1\right)+r_{1}=\left(t+1\right)k},y_{\left(t+1\right)k+1},y_{\left(t+2\right)k+2},\ldots ,y_{\left(n-2\right)k+\left(n-t-2\right)},y_{\left(n-1\right)k+r_{2}}\right\}, & \text{if}\;r_{1}>k-n+2, \end{array}$$

where *r*_1_ = 1, 2, …, *k*; *r*_2_ = 1, 2, …, *k*.

The sample mean based on the proposed systematic sampling scheme is given by:

$${\bar{y}}_{ndsy}=w_{1}{\bar{y}}_{1}+w_{2}{\bar{y}}_{2},$$ (3)

where

$${\bar{y}}_{1}=\{\begin{array}{ll} \frac{1}{n}\left(\sum_{l=0}^{n-2}y_{l\left(k+1\right)+r_{1}}+y_{\left(n-1\right)k+r_{2}}\right), & \text{if}\;r_{1}\leq k-n+2,\\ \frac{1}{n}\left(\sum_{l=0}^{t}y_{l\left(k+1\right)+r_{1}}+\sum_{i=1}^{n-t-2}y_{\left(t+i\right)k+i}+y_{\left(n-1\right)k+r_{2}}\right), & \text{if}\;r_{1}>k-n+2. \end{array},$$ (4)

$${\bar{y}}_{2}=y_{\left(n-1\right)k+r_{2}},$$ (5)

$$w_{1}=\frac{\left(n-1\right)k}{N},w_{2}=\frac{k}{N},w_{1}+w_{2}=1.$$

**Theorem**: Under the proposed sampling scheme, the sample mean can be written in the form of Horvitz-Thompson estimator ${\bar{y}}_{HT}$ suggested by Horvitz and Thompson [25] and is unbiased with variance:

$$Var\left({\bar{y}}_{ndsy}\right)=\frac{1}{N^{2}}\left[{\left(n-1\right)}^{2}k^{2}\left\{\frac{1}{k}\sum_{i=1}^{k}{\left({\bar{y}}_{1}-{\bar{Y}}_{1}\right)}^{2}\right\}+k^{2}\left\{\frac{1}{k}\sum_{i=1}^{k}{\left({\bar{y}}_{2}-{\bar{Y}}_{2}\right)}^{2}\right\}\right],$$

where,

${\bar{Y}}_{1}$: Mean of all (*n* − 1)*k* units of Set-1,

${\bar{Y}}_{2}$: Mean of all *k* units of Set-2.

**Proof**: By definition,

$$\begin{array}{ll} {\bar{y}}_{ndsy} & =\frac{\left(n-1\right)k}{N}{\bar{y}}_{1}+\frac{k}{N}{\bar{y}}_{2},\\ & =\frac{1}{N}\left(k\sum_{i\in s_{1}}y_{1i}+k\sum_{i\in s_{2}}y_{2i}\right), \end{array}$$ (6)

where *s*_1_ and *s*_2_ denote the samples drawn from Set-1 and Set-2 respectively.

$$\begin{array}{ll} {\bar{y}}_{ndsy} & =\left(\sum_{i\in s_{1}}\frac{y_{1i}}{1/k}+\sum_{i\in s_{2}}\frac{y_{2i}}{1/k}\right),\\ & =\frac{1}{N}\sum_{i\in s}\frac{y_{i}}{\pi_{i}}={\bar{y}}_{HT}, \end{array}$$ (7)

where ‘s’ denotes the total number of units selected in the sample.

Taking expectation on both sides of (6) yields:

$$E\left({\bar{y}}_{ndsy}\right)=\frac{\left(n-1\right)k}{N}E\left({\bar{y}}_{1}\right)+\frac{k}{N}E\left({\bar{y}}_{2}\right).$$ (8)

Now,

$$\begin{array}{ll} E\left({\bar{y}}_{1}\right) & =E\left(\frac{1}{n-1}\sum_{i=1}^{n-1}y_{1i}\right)=\frac{1}{n-1}\sum_{i=1}^{n-1}E\left(y_{1i}\right),\\ & =\frac{1}{n-1}\sum_{i=1}^{n-1}\left[\sum_{i\in S_{1}}y_{1i}\frac{1}{\left(n-1\right)k}\right],\\ & =\frac{1}{\left(n-1\right)k}\sum_{i\in S_{1}}y_{1i}={\bar{Y}}_{1}. \end{array}$$ (9)

Similarly,

$$E\left({\bar{y}}_{2}\right)=\frac{1}{k}\sum_{i\in S_{2}}y_{2i}={\bar{Y}}_{2},$$ (10)

where *S*_1_ and *S*_2_ denotes all units in Set-1 and Set-2 respectively.

Substituting (9) and (10) in (8) and simplification yields:

$$E\left({\bar{y}}_{ndsy}\right)=\bar{Y}.$$

Taking variance on both sides of (3) yields:

$$Var\left({\bar{y}}_{ndsy}\right)=\frac{{\left(n-1\right)}^{2}k^{2}}{N^{2}}Var\left({\bar{y}}_{1}\right)+\frac{k^{2}}{N^{2}}Var\left({\bar{y}}_{2}\right),$$ (11)

where

$$Var\left({\bar{y}}_{1}\right)=\frac{1}{k}\sum_{i=1}^{k}{\left({\bar{y}}_{1}-{\bar{Y}}_{1}\right)}^{2},$$ (12)

and,

$$Var\left({\bar{y}}_{2}\right)=\frac{1}{k}\sum_{i=1}^{k}{\left({\bar{y}}_{2}-{\bar{Y}}_{2}\right)}^{2}.$$ (13)

Substituting (12) and (13) in (11), the variance of ${\bar{y}}_{mdsy}$ is obtained as:

$$Var\left({\bar{y}}_{ndsy}\right)=\frac{1}{N^{2}}\left[{\left(n-1\right)}^{2}k^{2}\left\{\frac{1}{k}\sum_{i=1}^{k}{\left({\bar{y}}_{1}-{\bar{Y}}_{1}\right)}^{2}\right\}+k^{2}\left\{\frac{1}{k}\sum_{i=1}^{k}{\left({\bar{y}}_{2}-{\bar{Y}}_{2}\right)}^{2}\right\}\right].$$ (14)

**Remark 1**: Using Sen-Yates-Grundy approach suggested by Sen [26] and Yates and Grundy [27], the variance of ${\bar{y}}_{ndsy}$ can be written as:

$$Var\left({\bar{y}}_{ndsy}\right)=Var_{SYG}\left({\bar{y}}_{HT}\right)=\frac{1}{N^{2}}\left\{\frac{1}{2}\sum_{i=1}^{N}\sum_{\begin{array}{l} j=1\\ j\neq i \end{array}}^{N}\left(\pi_{i}\pi_{j}-\pi_{ij}\right){\left(\frac{y_{i}}{\pi_{i}}-\frac{y_{{}_{j}}}{\pi_{j}}\right)}^{2}\right\}.$$ (15)

**Remark 2**: The Sen-Yates-Grundy estimator for (15) is given by:

$$var\left({\bar{y}}_{ndsy}\right)=var_{SYG}\left({\bar{y}}_{HT}\right)=\frac{1}{N^{2}}\left\{\frac{1}{2}\sum_{i=1}^{n}\sum_{\begin{array}{l} j=1\\ j\neq i \end{array}}^{n}\left(\frac{\pi_{i}\pi_{j}-\pi_{ij}}{\pi_{ij}}\right){\left(\frac{y_{i}}{\pi_{i}}-\frac{y_{{}_{j}}}{\pi_{j}}\right)}^{2}\right\}.$$ (16)

The values of *π*_*i*_ and *π*_*ij*_ can be used from (1) and (2) in expression (15) and (16) to obtain the sampling variance of the mean and its estimator under the modified diagonal systematic sampling scheme. Moreover, it is to be noted that since the second order probabilities are zero for some pairs of units, so it is not possible to unbiasedly estimate $Var\left({\bar{y}}_{ndsy}\right)$. This is a common drawback of linear and systematic sampling, Subramani’s [8] method and the proposed method.

## 3. Linear trend

Let the *N* = *nk* = (*n* − 1)*k* + *k*, *k* ≥ *n* − 1 units of the finite population follow a linear trend. That is,

$$y_{i}=a+ib,\;\text{for}\;i=1,2,3,\ldots ,N.$$ (17)

The variance of the mean based on simple random sampling scheme in the case of linear trend is given by:

$$Var\left({\bar{y}}_{r}\right)=\left(k-1\right)\left(N+1\right)\frac{b^{2}}{12}.$$ (18)

The sampling variance of the mean in linear systematic sampling is given by:

$$Var\left({\bar{y}}_{sy}\right)=\left(k-1\right)\left(k+1\right)\frac{b^{2}}{12}.$$ (19)

The variance of the mean in diagonal systematic sampling is:

$$Var\left({\bar{y}}_{dsy}\right)=\left(k-n\right)\left[n\left(k-n\right)+2\right]\frac{b^{2}}{12n}.$$ (20)

The variance of the mean based on Subramani’s [8] modified systematic sampling scheme is given by:

$$Var\left({\bar{y}}_{msy}\right)=\left(\frac{{\left(n-1\right)}^{2}+1}{n^{2}}\right)\left(k-1\right)\left(k+1\right)\frac{b^{2}}{12}.$$ (21)

The variance of the sample mean in Subramani’s [13] optimal circular systematic sampling is given by:

$$Var\left({\bar{y}}_{ocss}\right)=\frac{\left(N-1\right)(N+1)b^{2}}{12n^{2}},\;\text{where}\;N=nk\pm 1.$$ (22)

Finally, the variance of the mean in the proposed method is obtained as:

$$Var\left({\bar{y}}_{ndsy}\right)=w_{1}^{2}Var\left({\bar{y}}_{1}\right)+w_{2}^{2}Var\left({\bar{y}}_{2}\right).$$ (23)

Since there are (*n* − 1)*k* units in Set-1, this implies putting *n* = *n* − 1 in (20) leads to:

$$Var\left({\bar{y}}_{1}\right)=\frac{\left(k-n+1\right)\left[\left(n-1\right)\left(k-n+1\right)+2\right]b^{2}}{12\left(n-1\right)}.$$ (24)

Also, there are *k* units in Set-2 and since the right hand side of (19) is independent of *n*, so

$$Var\left({\bar{y}}_{2}\right)=Var\left({\bar{y}}_{sy}\right)=\left(k-1\right)\left(k+1\right)\frac{b^{2}}{12}.$$ (25)

Using (24) and (25) in (23), the variance of ${\bar{y}}_{ndsy}$ in the case of linear trend is obtained as:

$$Var\left({\bar{y}}_{ndsy}\right)=\left\{\left(n-1\right)\left(k-n+1\right)\left[\left(n-1\right)\left(k-n+2\right)+2\right]+\left(k-1\right)\left(k+1\right)\right\}\frac{b^{2}}{12n^{2}}.$$ (26)

## 4. Efficiency comparison of the proposed systematic sampling with existing methods

If the units of population follow a linear trend, the proposed modified diagonal systematic sampling design is more precise than simple random sampling scheme if:

$$Var\left({\bar{y}}_{ndsy}\right)<Var\left({\bar{y}}_{r}\right).$$ (27)

Using (18) and (26) in (27) and on simplification, the condition reduces to:

$$\left(n-1\right)\left(k-n+1\right)\left[\left(n-1\right)\left(k-n+2\right)+2\right]<\left(k-1\right)\left[n^{2}\left(N+1\right)-\left(k+1\right)\right].$$ (28)

The proposed sampling scheme is more efficient than linear systematic sampling scheme if:

$$Var\left({\bar{y}}_{ndsy}\right)<Var\left({\bar{y}}_{sy}\right).$$ (29)

Using (19) and (26) in (29) and on simplification the condition reduces to:

(n−1)(k−n+1)[(n−1)(k−n+2)+2]<0. (30)

The proposed sampling scheme is more efficient than Subramani’s [8] modified linear systematic sampling scheme if:

$$Var\left({\bar{y}}_{ndsy}\right)<Var\left({\bar{y}}_{msy}\right).$$ (31)

Using (21) and (26) in (31) and simplification yields:

(k−n+1)[(n−1)(k−n+2)+2]<(n−1)(k−1)(k+1). (32)

The proposed sampling design is more precise than diagonal systematic sampling design if:

$$Var\left({\bar{y}}_{ndsy}\right)<Var\left({\bar{y}}_{dsy}\right).$$ (33)

Using (20) and (26) in (33) and after simplification, condition (33) reduces to:

(n−1)(k−n+1)[(n−1)(k−n+2)+2]+(k−1)(k+1)<n(k−n)[n(k−n)+2]. (34)

Eq (34) usually holds if *k* is more than twice the value of *n*. Table 3 shows the improvement in efficiency for different choices of *k* and *n*.

## 5. A numerical illustration using milk yield data

The milk yield data of S-19 brand of Sahiwal cows for 252 days from the date of calving was obtained from Pandey and Kumar [28]. From the daily observed milk yield of cows (in liters) as given in Pandey and Kumar [28], one can observe that with the passage of time, the milk yield decreases, leading to a linear trend in the data set.

The variances of different sampling schemes on the basis of milk yield data are given in Table 2. It clear that the proposed modified diagonal sampling scheme is more efficient than some of the available sampling designs including both the diagonal systematic sampling and Subramani’s [8] sampling scheme. It is worth noting that since the population size is *N* = 252 units and systematic sampling requires that *N* = *nk*, therefore, in order to make efficiency comparison possible, a few units were deleted from the population for some choices of *n* and *k* so that the value of *N* reconciles with *n* and *k*. For example, for *n* = 10 and *k* = 25, the last two units were deleted thus reducing the population size *N* = 250. In this case, *N* = 250 was used for the calculation of variance for each sampling scheme in order to make the comparison under identical conditions.

**Table 2:** Variances of different sampling schemes for milk yield data.

*n*	*k*	$Var\left({\bar{y}}_{r}\right)$	$Var\left({\bar{y}}_{sy}\right)$	$Var\left({\bar{y}}_{dsy}\right)$	$Var\left({\bar{y}}_{msy}\right)$	$Var\left({\bar{y}}_{ndsy}\right)$
83	3	1.7329	0.9154	2.0410	1.4508	0.8653
63	4	1.5051	0.7900	1.1436	1.0135	0.5180
49	5	1.0628	0.3826	0.7286	0.5673	0.3496
42	6	0.9291	0.2904	0.5042	0.4358	0.2604
35	7	0.7690	0.2781	0.3686	0.3025	0.2267
31	8	0.7157	0.2496	0.2812	0.2316	0.1807
28	9	0.6143	0.1301	0.2214	0.1814	0.1268
25	10	0.5243	0.1151	0.1785	0.1467	0.1097
22	11	0.5034	0.1093	0.1535	0.1249	0.0988
21	12	0.4632	0.1071	0.1230	0.1161	0.0923
19	13	0.4153	0.1060	0.1042	0.0983	0.0891
18	14	0.3613	0.0990	0.0896	0.0871	0.0856
16	15	0.3650	0.0930	0.0775	0.0716	0.0633

## 6. Conclusion

The variances of the mean based on simple random sampling, linear systematic sampling, diagonal systematic sampling, Subramani’s [8] modified systematic sampling, Subramani’s [13] optimal circular systematic sampling, and the proposed modified diagonal systematic sampling method for various choices of *n* and *k* have been presented in Table 3. The values of *n* and *k* have been chosen in such a way that *N* = *nk* and *k* ≥ *n* − 1. Since the constant *b*^2^ is a multiple in the variance of every sampling scheme, so *b* = 1 has been used to make comparison simpler. Efficiency comparison has been made for small sample sizes as well as for large sample sizes. It is worth mentioning that since Subramani’s [13] optimal circular systematic sampling requires that *N* = *nk* ± 1, so in order to make efficiency comparison possible with Subramani’s [13] procedure, the value of *N* = *nk* + 1 is used in the calculation of the variance in Table 3. For *N* = *nk* − 1, the computations of variance of Subramani’s [13] sampling scheme will be almost the same as those for *N* = *nk* + 1, so the values of the variance of Subramani’s [13] sampling scheme are presented only for *N* = *nk* + 1. It is clear that the suggested modified diagonal systematic sampling scheme is better than the other existing sampling schemes in terms of efficiency. This high gain in efficiency makes the proposed modified diagonal systematic sampling schemes more preferable than the existing sampling schemes in situations where the population units follow a linear trend.

**Table 3:** Variances of the mean under various sampling schemes.

*n*	*k*	$Var\left({\bar{y}}_{r}\right)$	$Var\left({\bar{y}}_{sy}\right)$	$Var\left({\bar{y}}_{dsy}\right)$	$Var\left({\bar{y}}_{msy}\right)$	$Var\left({\bar{y}}_{ocss}\right)$	$Var\left({\bar{y}}_{ndsy}\right)$
5	15	88.67	18.67	8.67	12.69	19.25	8.08
	20	159.92	33.25	19.25	22.61	34.00	16.26
	25	252.00	52.00	34.00	35.36	52.92	27.28
10	30	727.42	74.92	33.67	61.43	75.50	32.25
	40	1303.25	133.25	75.50	109.27	134.00	68.76
	50	2045.75	208.25	134.00	170.77	209.17	118.93
30	80	15806.58	533.25	208.61	498.89	533.78	207.38
	100	24758.25	833.25	408.72	779.55	833.89	399.38
	120	35709.92	1199.92	675.50	1122.59	1200.67	653.75
50	150	93137.42	1874.92	833.67	1801.42	1875.50	825.58
	200	165849.92	3333.25	1875.50	3202.59	3334.00	1838.75
	250	259395.75	5208.25	3334.00	5004.09	5209.17	3252.25
70	200	232183.25	3333.25	1408.64	3239.37	3333.81	1401.11
	250	363145.75	5208.25	2700.43	5061.57	5208.93	2668.78
	300	523274.92	7499.92	4408.88	7288.69	7500.71	4341.38
100	300	747524.92	7499.92	3333.67	7351.42	7500.50	3317.25
	400	1330033.25	13333.25	7500.50	13069.25	13334.00	7426.25
	500	2079208.25	20833.25	13334.00	20420.75	20833.25	13168.92
